# Mechanism of *Valeriana officinalis L.* extract improving atherosclerosis by regulating PGC-1α/Sirt3/Epac1 pathway

**DOI:** 10.3389/fphar.2024.1483518

**Published:** 2024-11-19

**Authors:** Bo Yao, Jingzhuo Ma, Qingzhi Ran, Hengwen Chen, Xuanhui He

**Affiliations:** ^1^ Guang’anmen Hospital, China Academy of Chinese Medical Sciences, Beijing, China; ^2^ School of Pharmaceutical Sciences, Changchun University of Chinese Medicine, Changchun, China

**Keywords:** *Valeriana officinalis L.* extract, atherosclerosis, PGC-1α/Sirt3/Epac1 pathway, mitochondrial damage, traditional Chinese medicine

## Abstract

**Objective:**

To investigate the protective effect of the of *Valeriana officinalis L.* extract on mitochondrial injury in AS mice and the underlying mechanism.

**Methods:**

Firstly, Ultra-High performance liquid chromatography-quadrupole time-of-flight mass spectrometer (UPLC / Q-TOF-MS) was proposed to explore the chemical composition of *Valeriana officinalis L.* extract. ApoE-/- mice were employed for in vivo experiments. The efficacy of *Valeriana officinalis L.* extract was detected by B-ultrasound, Biochemical, Oil Red O staining, HE staining and Masson staining analysis. The molecular mechanism of *Valeriana officinalis L.* extract in regulating mitochondrial energy metabolism for the treatment of atherosclerosis was elucidated after Monitoring System of Vascular Microcirculation *in Vivo* and transmission electron microscopy. Use the corresponding reagent kit to detect ACTH level, CHRNα1 level and ATP level, and measure the expression levels of PGC-1α, Sirt3, Epac1, Caspase-3, and Caspase-9 through real-time qPCR, and Western blot.

**Results:**

A total of 29 metabolites were newly discovered from KYXC using UPLC-MS. The drug had a significant positive effect on the growth of atherosclerotic plaque in mice. It also improved the microcirculation of the heart and mesentery, reduced the levels of CHOL, TG, and VLDL in the serum, and increased the levels of HDL-C to maintain normal lipid metabolism in the body. Additionally, it increased the levels of ATP, improved the ultrastructure of mitochondria to maintain mitochondrial energy metabolism, and increased the levels of T-SOD to combat oxidative stress of the organism. Furthermore, the drug significantly increased the mRNA and protein expression of PGC-1α and Sirt3 in aortic tissue, while decreasing the mRNA and protein expression of Epac1, Caspase-3, and Caspase-9.

**Conclusion:**

This study has verified that the extract of *Valeriana officinalis L.* is highly effective in enhancing atherosclerosis disease. The mechanism is suggested through the PGC-1α/Sirt3/Epac1 signaling pathway, which improves mitochondrial energy metabolism.

## 1 Introduction

Cardiovascular disease (CVD) is the leading cause of morbidity and mortality, affecting over 523 million people globally, and CVD mortality rates are predominated by atherosclerotic diseases (Nedkoff et al., 2023). Atherosclerosis (AS), a cardiovascular disease with a significant global mortality rate, has been a prominent focus of research in related fields for many years (Arnett et al., 2019). While medication treatment and surgical intervention can partially impede the progression of AS in clinical, cardiovascular and cerebrovascular diseases resulting from AS remain a significant global cause of mortality and disability (Marchio et al., 2019). Atherosclerosis is an essential clinical pathophysiological process characterized by endothelial damage and dysfunction leading to lipid deposition in the vascular wall. It involves various structural alterations in the vascular walls of large and medium-sized arteries, followed with the development of atherosclerotic plaques (Libby, 2021). This condition is typically manifested by the excessive accumulation of ox-LDL and intravascular hyperplasia (Jaganjac et al., 2020). Under microscopic examination, various pathological changes can be observed, including lipid deposition, smooth muscle cell proliferation, monocyte infiltration, and increased external matrix (Falk, 2006). These changes are strongly associated with the mechanisms of lipid metabolism disorder, inflammatory reaction, vascular endothelial cell dysfunction, and other mechanisms (Zhou and Wu, 2023). Thus, the occurrence and development of atherosclerosis can be effectively inhibited. By controlling lipid metabolism, reducing inflammatory reaction, and improving the function of vascular endothelial cells.

The ApoE^−/−^ mice is currently a widely used model for studying atherosclerosis. This is due to its ability to develop atherosclerotic lesions spontaneously, as well as its close resemblance to humans in terms of pathology and histology (Manea et al., 2020). Additionally, research has demonstrated that the progression of atherosclerosis in ApoE^−/−^ mice closely resembles type II hyperlipidemia in humans. These mice readily develop atherosclerotic plaques at specific locations, including the aortic root, lesser curvature of the aortic arch, major branches of the aorta, as well as branches of the pulmonary and carotid arteries (Haofei Han et al., 2019). The study included a group of ApoE^−/−^ mice who were fed a standard diet. ApoE^−/−^ mice that were given a typical diet experienced challenges in initiating AS lesions and developing AS plaques. The introduction of a diet high in fat expedited the onset and progression of AS, aligning with existing literature findings.

Peroxisome proliferator-activated receptor-γ coactivator (PGC-1α) is a transcription co activator in regulating metabolic processes in the body (Rius-Pérez et al., 2020). It does so by influencing the activity of various transcription factors involved in metabolic signal transduction, mitochondrial function, inflammation, oxidative stress, etc. (Pérez et al., 2019) Sirtuin 3 (Sirt3) is a type III histone deacetylase that relies on nicotinamide adenine dinucleotide (NAD^+^) for its function. It has a crucial role in regulating mitochondrial respiration, ROS clearance, lipid metabolism, fatty acid oxidation, and antioxidant activity (Lv et al., 2017). Epac, as an effector of cAMP, has been shown to protect myocardial cells from ischemic damage in the heart due to the absence of Epac1. Additionally, inhibiting the expression of Epac1 gene can suppress mitochondrial fission and ROS generation in vascular smooth muscle cells (Musheshe et al., 2022). Research has identified Sirt3 as the target gene of PGC-1α, and PGC-1α can greatly influence mitochondrial generation with inducing Sirt3 expression, and Giralt’s experiment suggests that an Epac1 signalosome may exist at the mitochondria (Giralt et al., 2011; Fazal et al., 2017). Previous research conducted using a high-fat diet mouse model found that PGC-1α can promote lipid metabolism and fatty acid oxidation (Weerawatanakorn et al., 2023). Thus, enhancing the expression of PGC-1α/Sirt3/Epac1 signaling pathway to improve lipid deposition and thus regulate mitochondrial energy metabolism is a promising method to promote the regression of atherosclerotic plaque.

*Valeriana officinalis L.* (Caprifoliaceae; *Valeriana officinalis L.* radix et rhizoma) is a perennial herbaceous plant belonging to the Caprifoliaceae family and the Valeriana genus. The “Quality Standards for Chinese and Ethnic Medicinal Materials in Guizhou Province” (2003 edition) includes it, and it is widely used as a traditional medicinal material in Jiangsu Province, Zhejiang Province, Hubei Province, Sichuan Province, Guizhou Province and other places in China. It often grows under forests, along ravines, and on high mountain slopes (Hao Zhang and Keyi, 2023). Its nature is spicy, sweet, and warm, and it is associated with the heart and liver meridians. It has functions such as clearing heat and stopping diarrhea, calming the heart and mind, regulating qi and relieving pain, and dispelling wind and dampness (Zhang, 2021). The investigation into the therapeutic application of the traditional Chinese medicinal *Valeriana officinalis L.* for cardiovascular diseases commenced in 1994. Our group isolated 65 metabolites from the roots and stems of *Valeriana officinalis L.* in the previous study (Chen et al., 2016; Chen et al., 2015). We subsequently carried out animal and cellular studies to confirm that certain metabolites have the ability to enhance myocardial ischemia. Clinically, it has been proved that the volatile oil of *Valeriana officinalis L.* has notable therapeutic properties in relieving angina symptoms and improving myocardial ischemia. Furthermore, it has been found to be more effective than metabolite salvia miltiorrhiza in improving angina symptoms, reducing the frequency of angina, shortening the duration of angina attacks, and no toxicity to liver, kidney, and hematopoietic tissues (Yang and Wang, 1994). Moreover, Zhu et al. (2016), discovered that the iridoids in valerian can significantly reduce the lipid biochemical indicators in the serum of hyperlipidemic rats, enhance the ability of lipid metabolism in rats, and thus reduce the incidence of atherosclerosis. However, the mechanism of *Valeriana officinalis L.* on atherosclerosis is not yet fully understood.

**FIGURE 1:**
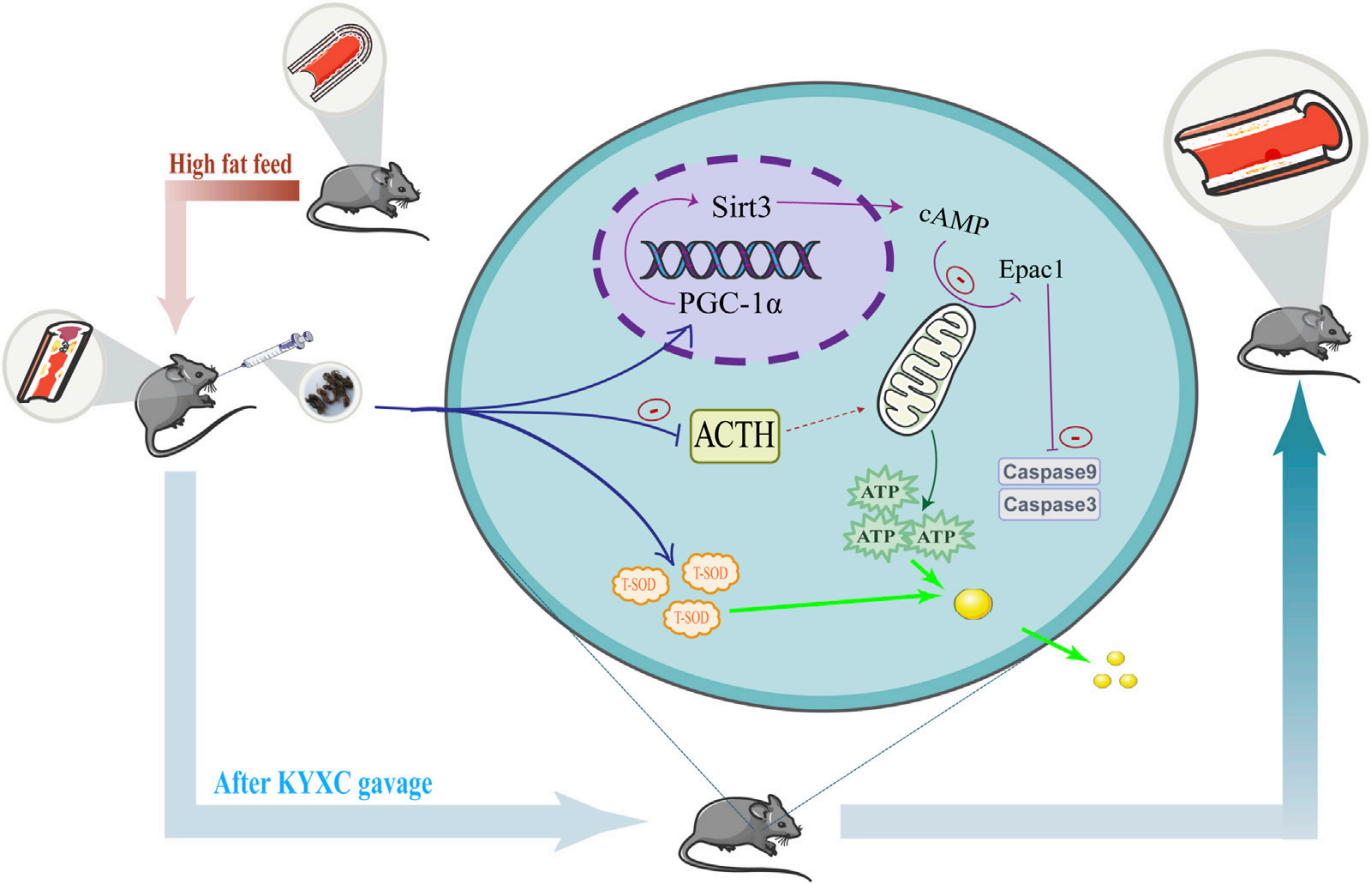
Mechanism diagram of the mechanism corresponding to KYXC improving plaque formation in AS mice.

This work aimed to explore the potential of *Valeriana officinalis L.* extract (KYXC) in improving AS plaque formation, and to uncover its precise molecular mechanism. The molecular mechanism is shown in Figure 1. After establishing an AS model in ApoE^−/−^ mice, it was found that KYXC can inhibit lipid production and aggregation, enhance ATP synthesis, and improve mitochondrial function. Regarding the mechanism, it is noteworthy that KYXC can activate the expression of PGC-1α and Sirt3, while reducing the expression levels of Epac1 and apoptotic proteins such Caspase-3 and Caspase-9. This leads to an enhancement in mitochondrial energy metabolism. The study has discovered and proven the effectiveness of utilizing *Valeriana officinalis L.* extract as a treatment for AS, which could potentially establish a novel approach for a new treatment method, providing innovative research ideas for better clinical treatment of AS.

## 2 Materials and methods

### 2.1 Preparation of KYXC

*Valeriana officinalis L.* (No. 01670385) sample has been deposited in the Specimen Museum of the School of Pharmacy, Guiyang University of Traditional Chinese Medicine (No. 55 Shidong Road, Guiyang City, Guizhou Province 550009, China). Medicinal herb information can be accessed through the Chinese Virtual Herbarium, a digital herbarium in China (https://www.cvh.ac.cn/index.php) Search. The *Valeriana officinalis L.* extract was obtained from Shaanxi Haiyisi Biotechnology Co., Ltd., with batch number HYS20221025. According to the identification of Deputy Chief Pharmacist Yang Hui and Chief Pharmacist Rong Lixin, it is *Valeriana officinalis L.*

The dried radix et rhizoma of *Valeriana officinalis L.* are weighed at 10 kg. The medicinal herbs of *Valeriana officinalis L.* were extracted by 10 times of water through heating reflux for twice, each time for 1.0 h. The extracts were mixed and concentrated under reduced pressure. Ethanol was added to bring the ethanol content to 70%. After 24 h, the extracts were dried in vacuum at 60°C. Finally, 1.8 kg of KYXC extract were obtained. The dry extract yielding rate of KYXC was 18.00%.

In addition, we conducted physical and chemical quality testing on the extract of *Valeriana officinalis L.,* confirming that it is a brown powder with a special odor and a fine powder that can pass 100% through 80 mesh. Among them, its ash content is 3.18%, heavy metal content ≤10 ppm, arsenic (As) content ≤2 ppm, lead (Pb) content ≤2 ppm, cadmium (Cd) content ≤0.2 ppm, mercury (Hg) content ≤0.1 ppm, all of which conform the Traditional Chinese Medicine--Determination of heavy metals in herbal medicines used in Traditional Chinese Medicine (ISO 18664:2015).

### 2.2 Drugs

The Rosuvastatin calcium tablets were purchased from AstraZeneca Pharmaceuticals (China) Co., Ltd., with the national drug approval number HJ20160545 and batch number 503778. All of the aforementioned drugs have undergone inspection and are now being utilized.

### 2.3 Main reagents and antibodies

Mass spectrometry grade solvents (including methanol, acetonitrile, and formic acid) were purchased from Thermo Fisher Scientific Co., Ltd. Proteintech provided PGC-1α antibody mouse clonal antibody, Sirt3 antibody rabbit clonal antibody, Caspase-9 antibody mouse clonal antibody, and Caspase-3 antibody mouse clonal antibody. Gene Tex Company supplied Sirt3 antibody rabbit clonal antibody. Jackson supplied Epac1 HRP × Polyclonal Goat Anti-Rabbit IgG and HRP × Polyclonal Goat Anti-Mouse IgG.

### 2.4 Animal model and treatment

A cohort of 70 SPF grade C57BL/6J mice and ApoE^−/−^ mice, aged 8–10 weeks, male, weighing 22–26 g, were provided from Beijing Weitong Lihua Experimental Animal Technology Co., Ltd., with license number SCXK (Beijing) 2021-0006. They were raised in the SPF level animal laboratory of Guang’ anmen Hospital, Chinese Academy of Traditional Chinese Medicine, under license number SYXK (Beijing) 2014-0041. The optimal temperature for breeding is between 22°C–25°C, while the relative humidity should be maintained at 55%–70%. It is recommended to provide a 12-hour light-dark cycle and ensure that the animals have unrestricted access to food and water. The high-fat feed was purchased from Beijing Huafukang Biotechnology Co., Ltd., with certificate number SCXK (Beijing) 2019-0008. The high fat feed is composed of 78.85% basic feed, 21% fat, and 0.15% cholesterol, all of which have undergone sterilization using cobalt-60 beam irradiation. Finally, mice were euthanized at the end of the assay.

The mice were categorized into several groups: a control group (CG), a model group (MG), a blank group (BG), a rosuvastatin group (RG), a low-dose KYXC group (KG-L), a medium dose KYXC group (KG-M), and a high-dose KYXC group (KG-H). Each group consisted of 10 mice. The CG group utilized C57BL/6J normal mice, while the other groups employed ApoE^−/−^ mice of the same strain. With the exception of the CG group and the BG group, the remaining groups were administered a high-fat diet for a duration of 24 weeks in order to induce the development of an atherosclerosis model in mice. During the modeling period, a total of 3 mice were randomly selected from each group to undergo testing for the formation of aortic plaque every 4 weeks. A substantial difference (*P* < 0.05) suggested the successful establishment of the atherosclerosis mouse model. Following successful modeling, all groups were administered normal feed. The RG group received a dose of 0.05 g kg^−1^ d^−1^ via gavage, while the KYXC group received doses of 0.10, 0.21, and 0.43 g kg^−1^ d^−1^ via gavage. The CG, MG, and BG groups were given an equivalent volume of distilled water. This treatment regimen was maintained for a duration of 6 weeks.

### 2.5 UPLC/Q-TOF-MS analysis

A chemical composition analysis of the extract obtained from *Valeriana officinalis L.* using UPLC/Q-TOF-MS technique was carried out as below. The chromatographic settings included the use of acetonitrile (B) mixed with 0.1% formic acid water (A) as the mobile phase. The flow rate was set at 0.4 mL⋅min^−1^. The sampler temperature was maintained at 10°C. The sample size used was 2 µL. The temperature of the column is 40°C, and the range of wavelengths detected by the UV is from 190 nm to 500 nm. The MS conditions comprise of the electric spray ion source (ESI), both positive and negative ion detection modes, full scan scanning mode, and a scanning range of 50–1500 Da. Parameters of the ion source include a temperature of 120°C, nitrogen gas used as both the carrier gas and desolvent gas, a cone hole gas flow rate of 50 L/h, a desolvent gas temperature of 450°C, a desolvent gas flow rate of 800 L/h, and a cone hole voltage of 40 V. The capillary voltage is set at +3.0 KV in the positive ion mode and −2.5 KV in the negative ion mode. The gas used for collision induced dissociation is argon with a purity of 99.95%. The collision energy is 6 eV for low collisions and ranges from 12 to 45 eV for high collisions. The samples underwent testing using the 2,998 ultra-high performance liquid chromatography and Xevo G2-S Q-TOF high-resolution time-of-flight mass spectrometer, manufactured by Waters in the United States. The chromatographic column used is a Waters column with dimensions of 250 mm × 4.6 mm and a particle size of 5 μm. The extraction solvent consists of 50% methanol, and the recommended extraction duration is 30 min.

### 2.6 B-ultrasound analysis

Monitor the development of plaques in AS mice according on the timeline specified in the experimental design. The mouse’s front chest was shaved for skin preparation, and put into the small animal anesthesia machine for anesthesia. Following effective administration of anesthesia, it was fixed on the operating platform in a supine position. Aortic ultrasound and cardiac ultrasound were then conducted to examine the development of atherosclerotic plaque.

### 2.7 Biochemical analysis

Following a 6-week period of administration, blood samples were collected from the eyeball of mice. The levels of blood lipid indicators in the serum of mice, including CHOL, TG, LDL-C, and HDL-C, were then measured using a fully automated biochemical analyzer.

### 2.8 Microvascular analysis

Following a 6-week period of treatment, a single mice was randomly chosen from each group to undergo anesthesia. Subsequently, the heart and mesenteric microvasculature were observed by Monitoring System of Vascular Microcirculation *in Vivo* (OCTA). The OCTA algorithm is used for the extraction of three-dimensional microvascular signals. The acquired data is then extracted using vResolve software for vascular signal extraction, then picture synthesis is performed using Reconstruction UI software.

### 2.9 Oil red O staining analysis

Following a 6-week period of medication intervention, samples were collected after a 12-hour fasting period. Separate and remove the blood vessels in the general aortic region, fix them for a duration of 24 h, and subsequently dissect them open. Begin by immersing them in a 60% isopropanol solution for a duration of 3 s. Next, proceed to stain them using an oil red O staining solution at a temperature of 37°C in a dark environment for 60 min. Finally, remove them after the staining process is complete. Submerge them in a solution of 60% isopropanol to distinguish red adipose plaques in the inner space and colorless in other regions. Then, eliminate the blood vessels and use filter paper to eliminate any remaining water. Examine the entirety of the plaque formation in the aorta using a microscope, collect images and analyze the percentage of gross oil red fat area using Image Pro plus 6.0 software.

Obtain a specimen of aorta tissue, encase it in OCT, and slice it using a frozen sectioning machine with a thickness of 20 μm. The process involves staining, sealing, and observing the lipid staining in Aortic endothelial cells using Oil Red O staining method under the microscope.

### 2.10 HE and masson staining analysis

After a duration of 6 weeks, samples were collected and then embedded in paraffin to preserve the heart tissue specimen. Subsequently, the tissue was sectioned. The tissue was sliced into sequential sections measuring 4 μm in thickness. These sections were then submitted to HE and Masson staining in order to observe any pathological and morphological alterations associated with the heart. Images were acquired for analysis.

### 2.11 Mitochondrial functional analysis

Following a 6-week period of delivery, a sample of aorta tissue measuring roughly 1 mm^3^ was collected and subjected to a series of procedures including fixation, dehydration, infiltration, embedding, slicing, and staining. The prepared sample was then examined under a transmission electron microscope for observation. Collected images were subjected to analysis.

### 2.12 Serum index assay

After a duration of 6 weeks, adhere to the directions provided by the reagent kit to measure the concentrations of ACTH, CHRNα1, and ATP in the serum of mice.

### 2.13 Analysis of antioxidant levels

Perform a dissection on the cardiac tissue of mice on a platform made of ice. Subsequently, store the dissected tissue in a freezer set to a temperature of −80°C. Weigh the heart tissue and add corresponding reagents, grind it into tissue homogenate, centrifuge at 12,000 rpm for 15 min, and take the supernatant. Perform T-SOD activity detection in tissue supernatant following the directions provided with the reagent kit.

### 2.14 RT-qPCR analysis

Mice aorta and cardiac tissues were subjected to RNA extraction using Trizol reagent. Following reverse transcription of RNA into complementary DNA (cDNA) using a reverse transcription kit, a real-time quantitative PCR (qPCR) kit was employed to identify the presence of PGC-1α, Sirt3, Epac1, Caspase-9, and Caspase-3 mRNA in the aorta and heart tissue using a real-time quantitative PCR apparatus. Each reaction is replicated three times. The RT-qPCR primers are listed in Table 1.

**TABLE 1:** Primer sequence.

Gene name	Forward primers (5′-3')	Reverse primer (5′-3')
PGC-1α	AAC​TGC​AGA​TTT​GAT​GGA​GCT​AC	CAT​GTA​GAA​TTG​GCA​GGT​GGA
Sirt3	CTG​GAT​GGA​CAG​GAC​AGA​TAA​GA	GTT​GTG​GTC​TGG​TTC​ATG​TTT​GT
caspase-9	GCC​CCT​CAC​AGT​TCA​GAA​TGG	GCC​CAC​AAC​TGC​ATG​TGT​AGA
caspase-3	GCT​TCC​TTG​TTG​CAC​AAA​GAA	CAT​CAC​ATG​CTG​CAT​TCA​CTT​AG
Epac1	CGG​TGG​TCT​CAG​AGG​AGT​TGT​A	GAG​ATA​GAT​GGT​GGG​TGT​CTG​CT
GAPDH	CAA​GCT​CAT​TTC​CTG​GTA​TGA​CA	TCT​CTT​GCT​CAG​TGT​CCT​TGC​T

### 2.15 Western-blot analysis

Solidify To prepare the aortic tissue, first grind it. Then, take a portion of the ground tissue and add cold protease inhibitors and lysate. Next, break and grind the tissue using a tissue grinder. After that, centrifuge the mixture and collect the supernatant. Measure the protein concentration using the BCA protein quantification kit’s instructions. Finally, adjust the protein concentration using RIPA. Conduct Western Blot studies following the denaturation of the protein solution. Prepare the separation gel and concentration gel according to the molecular weight of the target protein. Employing the wet transfer technique for film transfer. Once the membrane transfer is finished, proceed to seal it for a duration of 30 min. Dilute the primary antibodies PGC-1α, Sirt3, Caspase-9, Caspase-3, and Epac1 in the respective ratios of 1:10000, 1:2000, 1:10000, 1:2000, and 1:1000. Incubate the diluted antibodies overnight at a temperature of 4°C. The secondary antibody chosen was goat anti-mouse IgG (H + L) HRP 1:10000. It was incubated at room temperature, in the absence of light, and on a shaking bed for a duration of 2 h. Position the chemiluminescence apparatus for automated exposure and conduct quantitative analysis utilizing ImageJ.

### 2.16 Statistical analysis

The grade data were analyzed using SPSS 27.0 software. The statistical analysis was conducted using GraphPad software version 6. The results were reported as the mean plus or minus the standard deviation. Comparisons involving more than two groups were conducted using one-way analysis of variance (ANOVA) with Bonferroni *post hoc* testing to evaluate the differences among the groups. A *p*-value of less than 0.05 was used to determine statistical significance.

## 3 Results

### 3.1 KYXC chemical metabolites analyzed by UPLC/Q-TOF-MS

**FIGURE 2:**
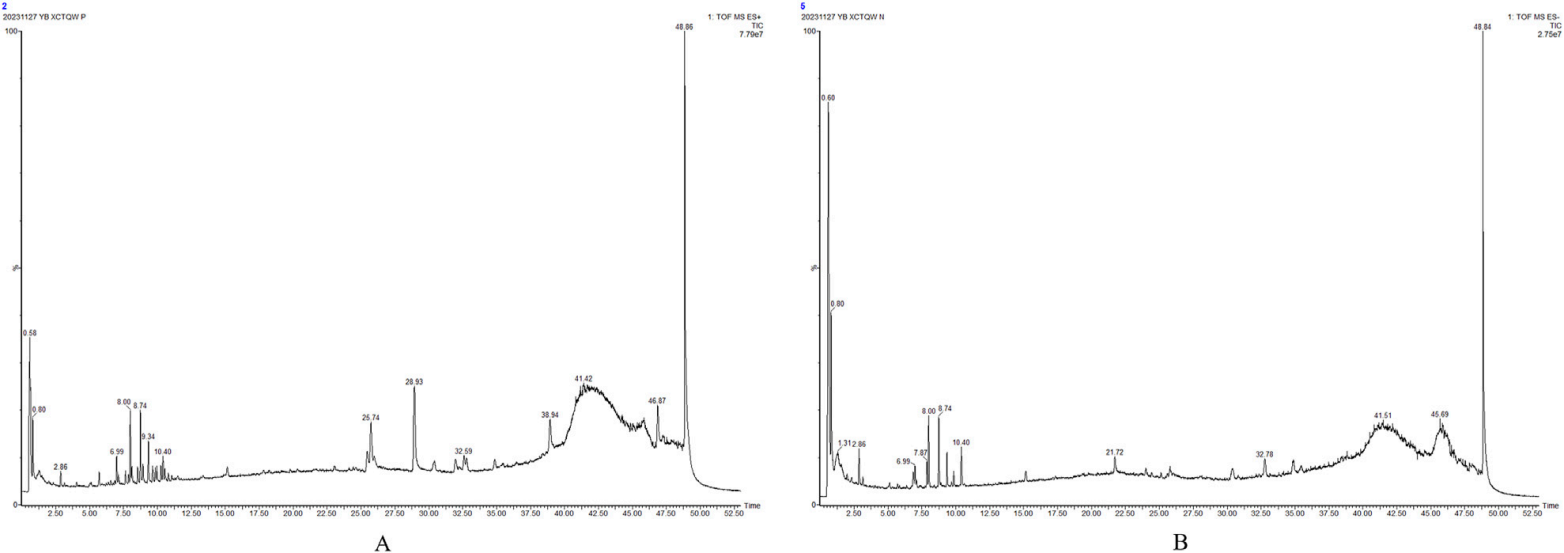
BPC of KYXC in positive ion mode. **(A)** BPC of KYXC in negative ion mode **(B)**.

This study initially organized the pertinent material on the chemical makeup of KYXC using literature and related databases. The chemical composition information of the traditional Chinese medication KYXC was compiled by conducting a comprehensive search of domestic and international literature, as well as relevant chemical databases like Scifinder, Pubmed, ChemicalBook, CNKI, etc. By utilizing chromatographic peak separation as a measure, the elution parameters were fine-tuned, and the ultimate analytical conditions were established. Under these circumstances, excellent chromatographic separation and a significant mass spectrometry response were achieved. Figures 2A, B depict the Basic Peak Graph (BPC) of positive and negative ions, respectively. The metabolites can be found in Table 2, 3. It is evident that a total of 29 substances were identified. Volvalerenic A is the primary metabolite among them.

**TABLE 2:** List of Constituent Analysis Metabolites for KYXC positive Ion Mode, including Chemical Abstracts Service Registry Numbers (CAS No.) and molecular weights (MW). Sorted alphabetically by acronym.

*Number*	*metabolite name*	*Formula*	*CAS*	*Adducts*	*Extraction Mass(Da)*	*Found At Mass* (*Da*)	*Mass error (mDa)*	*Observed RT (min)*	*Response*	*type*
1	1-hydroxy-1,11,11-decahydrocyclopropaneazulene-10-one	C14H22O2	—	+K	261	261.127	1.8	2.62	2,007	Magnolianes
2	3-caffeoylquinic acid methyl ester	C17H20O9	123483-19-2	+Na	368.34	391.0954	−4.5	5.75	1,834	Phenylacrylic acids
3	3β-Hydroxy-5α,8α- epidioxyergosta-6,22-diene	C29H50O2	—	+K	—	469.3429	−1.3	37.39	8,633	Triterpenes
4	8-Hydroxypinoresinol_1	C20H18O7	81426-17-7	+NH4	374.38	388.1345	−4.6	9.19	708	Lignan class
5	Benzoic acid	C7H6O2	65-85-0	+Na	122.12	145.0225	−3.5	3.15	1,058	Aromatic derivatives
6	lariciresinol-4-O-β-D-glucopyranoside	C26H34O11	143663-00-7	+H	522.54	523.2165	−0.8	9.57	777	Lignan class
7	oleanolic acid	C30H48O3	508-02-1	+Na	456.7	479.3446	−4.9	34.13	911	Triterpenes
8	olivil-4-O-β-D-glucopyranoside	C26H34O12	56440-73-4	+Na	538.55	561.1921	−2.2	4.2	1,521	Lignan class
9	Protocatechualdehyde	C7H6O3	139-85-5	+H	138.12	139.0433	4.3	0.82	1,060	Aromatic derivatives
10	Stearic acid	C18H36O2	57-11-4	+H	284.48	285.2743	−4.5	41.13	5,695	Carboxylic acids
11	Stigmastere-3,7-dione	C29H48O2	—	+H	—	429.3692	−3.5	42.9	1,2526	Triterpenes
12	stigmasterol	C29H48O	83-48-7	+H	412.7	413.373	−4.8	36.75	1,887	Triterpenes
13	Valtrate	C22H30O8	18296-44-1	+H	422.47	423.1973	−4	32.15	2,104	Cycloene ether terpenes
14	Volvalerenal K	C20H34O4	—	+Na	—	361.2317	−3.2	32.6	2,419	Magnolianes
15	Volvalerenl B	C15H20O3	—	+H	—	249.1439	−4.7	15.09	4,617	Gemaranes

**TABLE 3:** List of Constituent Analysis Metabolites for KYXC negative Ion Mode, including Chemical Abstracts Service Registry Numbers (CAS No.) and molecular weights (MW). Sorted alphabetically by acronym.

*Number*	*metabolite name*	*Formula*	*CAS*	*Adducts*	*Extraction Mass(Da)*	*Found At Mass* (*Da*)	*Mass error (mDa)*	*Observed RT (min)*	*Response*	*type*
1	(+)-Piresil-4-O-beta-D-glucopyraside	C26H32O11	69251-96-3	-H, +HCOO	520.53	519.1864	−0.8	7.33	4,233	Lignan class
2	4-Hydroxybenzoic acid	C7H6O3	99-96-7	-H	138.12	137.0254	1	2.5	692	Aromatic derivatives
3	4-Hydroxycinnamic acid	C9H8O3	7400-08-0	-H	164.16	163.0399	−0.1	4.59	854	Aromatic derivatives
4	Acacetin	C16H12O5	480-44-4	-H	284.26	283.0608	−0.4	10.41	24235	Flavonoids
5	caffeic acid	C9H8O4	331-39-5	-H	180.16	179.0351	0.1	3.28	1375	Aromatic derivatives
6	Ferulic Acid	C10H10O4	1135-24-6	+HCOO	194.18	239.0565	0.4	3.15	708	Aromatic derivatives
7	Pinorespiol	C20H18O8	4263-88-1	+Cl	358.39	421.0745	4.9	2.87	6,659	Lignan class
8	quercetin	C15H10O7	117-39-5	+HCOO	302.24	347.0426	1.7	15.16	5,533	Flavonoids
9	Rutin	C27H30O16	153-18-4	-H	610.52	609.145	−1.1	5.91	1528	Flavonoids
10	Sucrose	C12H22O11	57-50-1	-H	342.3	341.1084	−0.5	0.89	3,517	Carbohydrates
11	ursolic acid	C30H48O3	77-52-1	-H, +HCOO	456.71	455.3524	−0.7	30.36	116232	Triterpenes
12	Volvalerenal D	C15H22O4	—	-H	—	265.1473	2.8	42.52	33387	Gemaranes
13	Volvalerenic A	C15H22O2	—	-H	—	233.1542	−0.5	23.07	9,828	Gemaranes
14	Volvalerenl C	C16H22O4	—	-H	—	277.1439	−0.7	25.48	12780	Gemaranes

### 3.2 KYXC reduces the formation of aortic plaques in mice

**FIGURE 3:**
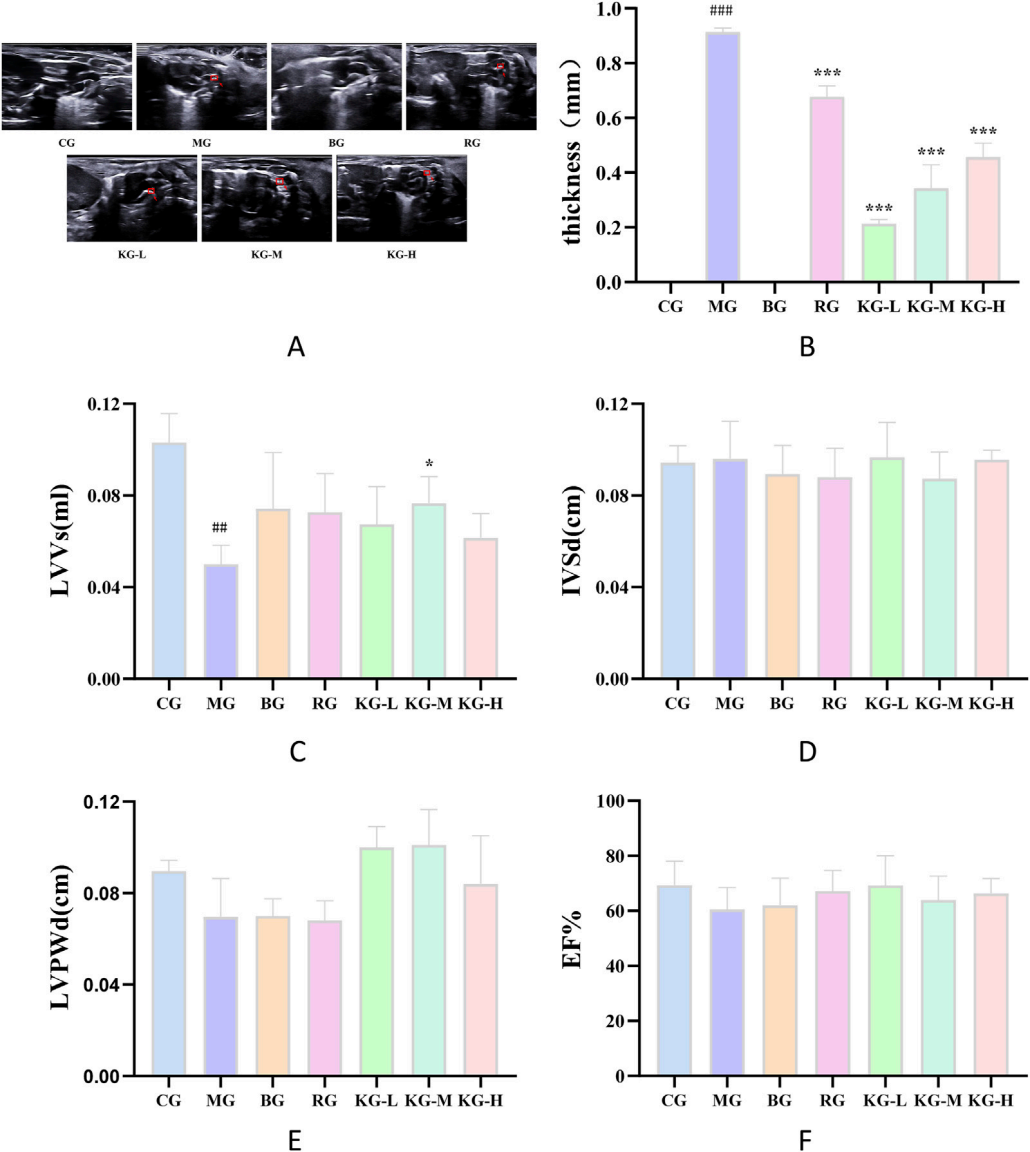
The effect of KYXC on mouse aortic plaques (The position indicated by the arrow in the figure is atherosclerotic plaque) **(A)** Effect of KYXC on Atherosclerotic Plaque in mice. **(B)** The Effect of KYXC on Mouse Cardiac Function. **(C–F)** (Compared with the CG group, #*P* < 0.05, ## *P* < 0.01, ### *P* < 0.001; Compared with the MG group, * *P* < 0.05, ** *P* < 0.01, *** *P* < 0.001).

Figures 3A, B demonstrate that mice in the MG group, after being fed a high-fat diet, developed many atherosclerotic plaques in both the aortic arch and abdominal aorta. This indicates that the modeling process was successful. When comparing the CG group to the MG group, the mice in the MG group exhibited noticeable plaque and an irregular aortic wall, with a significant distinction (*P <* 0.01). Additionally, when comparing the MG group to each treatment group, the atherosclerotic plaques were decreased to varying extents, and the difference was substantial (*P <* 0.001).

Cardiac remodeling and alterations in heart function are strongly associated with modifications in left ventricular volume (LVVs), interventricular septal thickness (IVSd), left ventricular posterior wall thickness (LVPWd), ejection fraction (EF), and other markers. Thus, these markers are frequently employed to assess heart function and can precisely depict cardiac function. According to Figures 3C–F, the LVVs of the MG group increased significantly compared to the CG group (*P <* 0.01). Additionally, the LVVs of the KG-M group were significantly reduced compared to the MG group *(P <* 0.05). Furthermore, the statistical analysis revealed that there was no significant impact on the markers of IVSd, LVPWd, EF, and other parameters. This suggests that the current condition of AS has not resulted in substantial alterations in the functioning of the heart.

### 3.3 KYXC improves blood lipid abnormalities in mice

**FIGURE 4:**
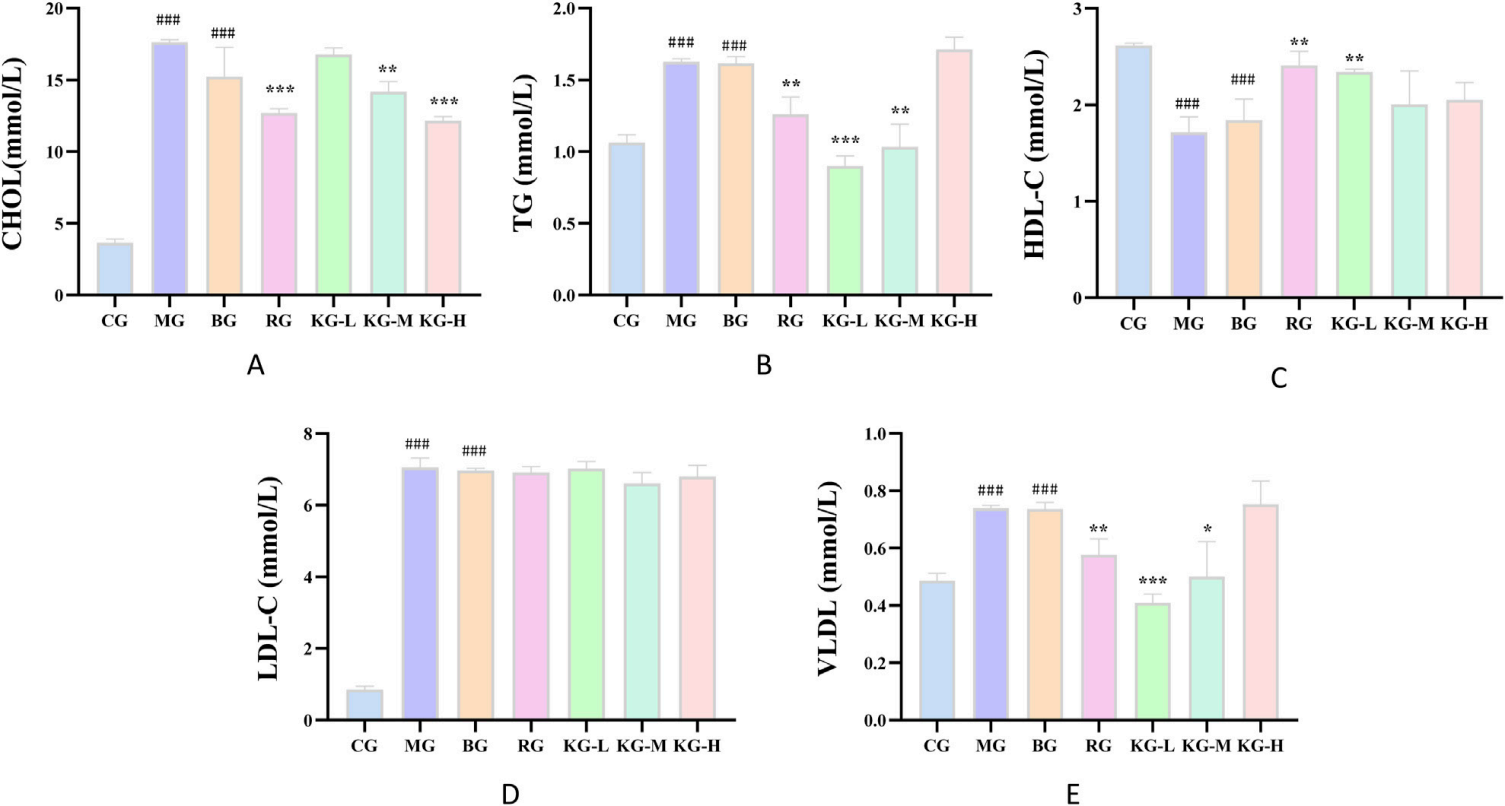
The effect of KYXC on blood lipids in AS mice **(A-E)**. (Compared with the CG group, # *P* < 0.05, ## *P* < 0.01, ### *P* < 0.001; Compared with the MG group, * *P* < 0.05, ** *P* < 0.01, *** *P* < 0.001).

The serum lipid levels in each group of mice were detected and the findings are presented in Figure 4. The MG group exhibited a considerable increase in the levels of CHOL, TG, VLDL, and LDL-C compared to the CG group (*P <* 0.001). Conversely, the HDL-C levels dramatically dropped (*P <* 0.001). Similarly, the BG group also showed a significant increase in all indicators (*P <* 0.001). The CHOL content in the KG-M, KG-H, and RG groups was dramatically decreased compared to the MG group (*P*< 0.001). Additionally, the TG and VLDL content in the RG, KG-L, and KG-M groups were reduced to varying degrees (*P <* 0.01). The HDL-C levels in the RG and KG-L groups showed a substantial rise (*P <* 0.01). The LDL-C levels in each treatment group declined to different extents, although there was no statistically significant difference.

### 3.4 KYXC promotes the formation of microvessels in mice

**FIGURE 5:**
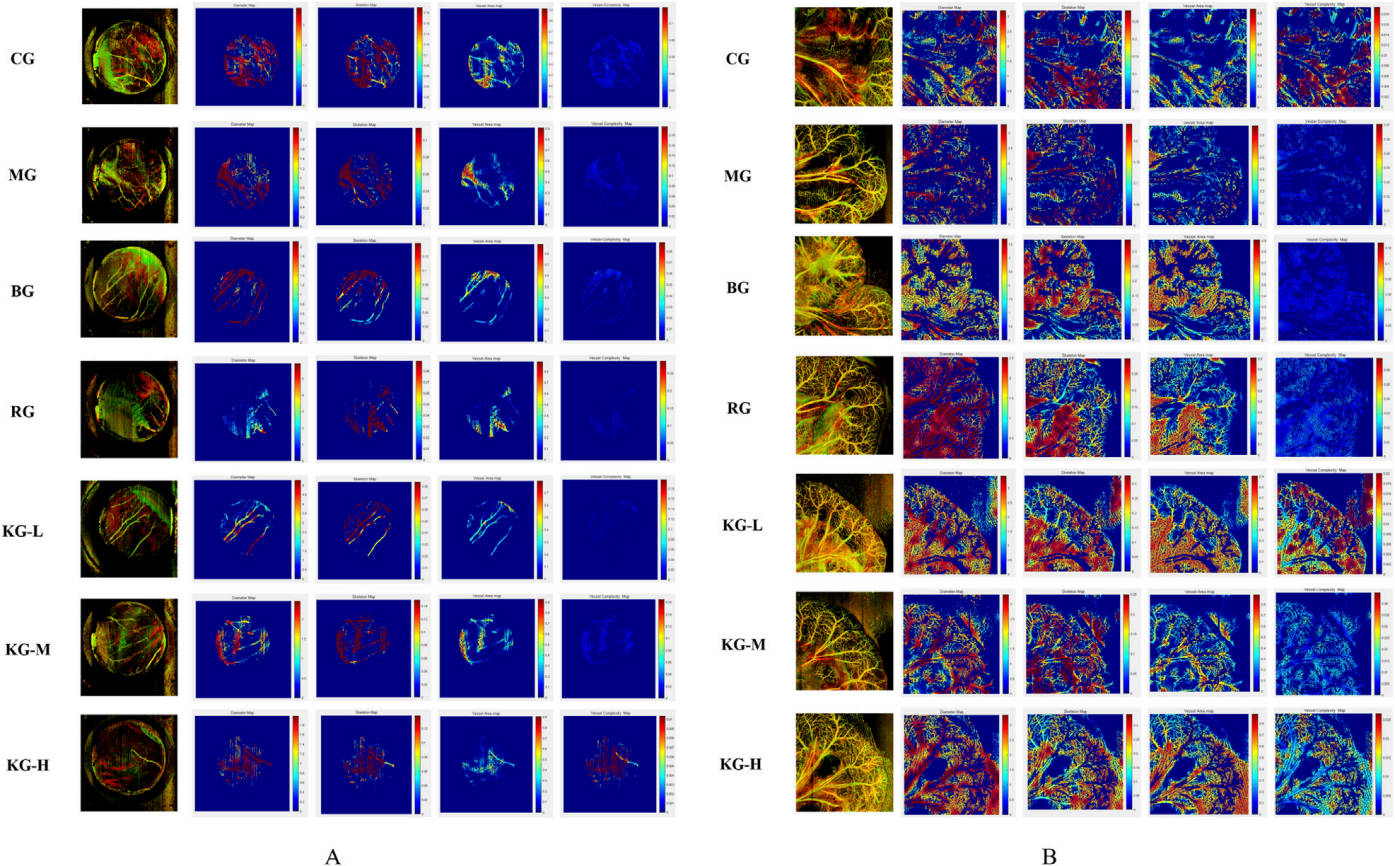
The effect of KYXC on Mouse Cardiac Microcirculation. **(A)** (Scanning Range 0.6 cm* 0.6 cm; Image Pixels 800 * 800) The effect of KYXC on intestinal and mesenteric microcirculation in mice. **(B)** (scanning range 1 cm * 1 cm; image pixels 900 * 900).

The Micro VCC (Optoprobe, United Kingdom) vascular microcirculation monitoring equipment can be utilized along with OCTA (optical coherence tomography angiography) mode scanning in the OPTO-III scanning program to acquire three-dimensional vascular data of the sample. The analysis software can be utilized to analyze the enhancement of microcirculation in the sample with greater intuitiveness and precision. The picture displays the following values from left to right: Vessel Perimeter Index (VPI), Vessel Diameter Index (VDI), Vessel Skeleton Density (VSD), Vessel Area Density (VAD), and Vessel Complexity Index (VCI). The color corresponds to the magnitude of blood flow, with red indicating the highest and blue indicating the lowest. VPI is a measure of the relationship between the circumference of blood vessels and the total area of an image. VDI is a measure of the relationship between the area of blood vessels and their length. VSD is a measure of the relationship between the length of blood vessels in an image and the total area of the image. VAD is calculated by comparing the area of blood vessels to the total area of the image. The formula for calculating VCI is VCI = P2/(4π*A), where *P* represents the circumference of the blood vessel and A represents the area of the blood vessel. Figure 5A depicts the microcirculation of the heart. The results indicate that the MG group exhibits varied degrees of reduction in VPI, VDI, VSD, VAD, and VCI compared to the CG group. Following KYXC therapy, the microcirculation coefficients of each group exhibited varied degrees of improvement, with KG-M and KG-H showing particularly notable enhancements. Figure 5B illustrates the microcirculation of the mesentery, which indirectly reflects the intestinal absorption of mice. The results indicate that the MG group shows a reduction in VP I, VDI, VSD, VAD, and VCI compared to the CG group. Following KYXC treatment, the microcirculation must be enhanced to different extents, particularly in the KG-L and KG-H groups.

### 3.5 KYXC reduces lipid droplet formation on the aortic wall

**FIGURE 6:**
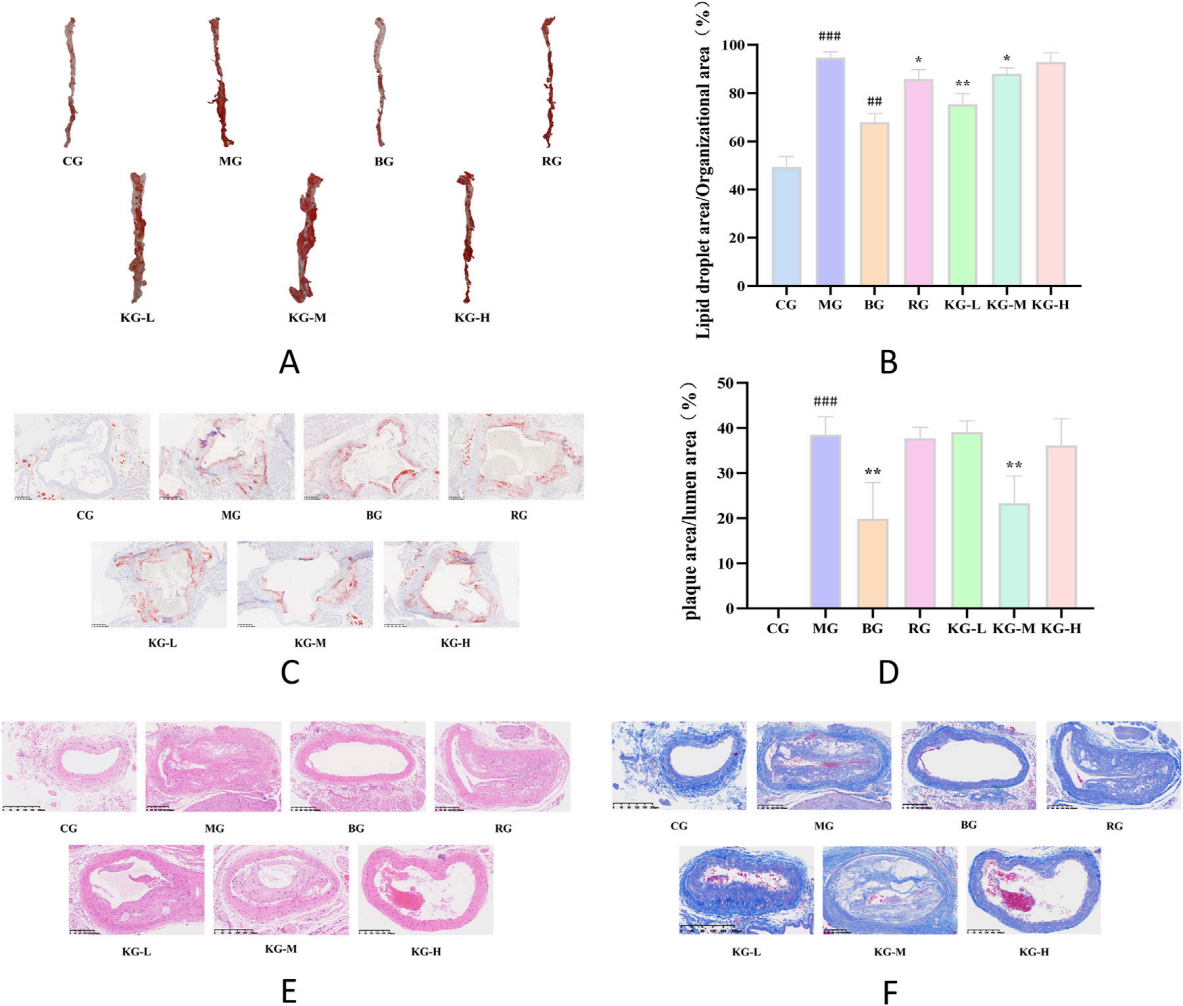
Oil Red O staining of the aorta of mice in each group. **(A)** The effect of KYXC on the ratio of aortic lipid droplet area to total tissue area in mice. **(B)** Red oil O staining of aortic roots in each group of mice. **(C)** (Scale bar: 200 μm) The effect of KYXC on the ratio of aortic plaque area to luminal area in AS mice. **(D)** HE staining of aortic roots in each group of mice. **(E)** Masson staining of aortic roots in each group of mice. **(F)** (Compared with the CG group, # *P* < 0.05, ## *P* < 0.01, ### *P* < 0.001; Compared with the MG group, * *P* < 0.05, ** *P* < 0.01, *** *P* < 0.001).

Figures 6A, B demonstrate that the red color observed on the aorta of mice, following staining with oil red O, corresponds to the area occupied by AS plaques. The aorta wall of mice in the CG group is translucent and has a smooth texture, without any noticeable accumulation of red lipid plaques. The study found no notable disparity in the inner lining of the aorta between the BG group and the CG group. This suggests that a regular diet did not have a substantial impact on the development of aortic plaques in ApoE^−/−^ mice (Figure 6C). In comparison to the CG group, the MG group had a significant accumulation of elevated red lipid deposits on the primary aortic vessel wall. These deposits were clearly demarcated from the vessel wall. In addition, the MG group had a significantly higher percentage of AS plaque area compared to the CG group (*P* < 0.001). After administering a low-dose KYXC, the plaque burden was significantly reduced, and the percentage of plaque area to total vascular area was significantly lower in the MG group (*P* < 0.01). Furthermore, the treatment effect was superior to that in the RG group (Figure 6B). As depicted in Figure 6D, the area of the aortic inner wall plate in the MG group expanded significantly (*P <* 0.001) compared to the CG group, and there was a substantial accumulation of lipids on the inner wall of the lumen. In each treatment group, lipid deposition was reduced to varying extents compared to the MG group, with the KG-M group showing particularly noticeable reduction.

### 3.6 KYXC improves pathological status of mice aortic tissue

Figures 6E, F display the results of HE and Masson staining of mice aortic tissue. The HE staining resulted in blue staining of the nuclei on the aorta wall, while the cytoplasm, muscle fibers, collagen fibers, and red blood cells were stained in different shades of red. On the other hand, Masson staining specifically dyed the collagen fibers in the aortic wall blue. The histological architecture of the inner wall of the aorta in the CG group is characterized by normalcy, integrity, and orderly arrangement. The inner, middle, and exterior membrane layers are distinctly observable, exhibiting well-defined borders and multiple layers of flexible membranes. Collagen deposition is negligible and there is no apparent formation of plaques. In the aorta of the MG group, the arrangement of smooth muscle cells is disorganized, with a notable thickening of the innermost layer, widespread lipid plaques, and considerable infiltration of inflammatory cells. There is a notable accumulation of collagen fibers in the connective tissue of the heart muscle, characterized by a disorganized pattern of strips or networks; The RG, KG-L, KG-M, and KG-H groups had a considerably improved pathological structure compared to the MG group. Additionally, there was a varied degree of reduction in AS plaques. There was a decrease in the severity of aortic intimal hyperplasia, as well as a significant reduction in the number of inflammatory cells. Simultaneously, the process of collagen deposition among the cells of the aorta wall was also enhanced to different extents, with KG-H showing the most notable improvement.

### 3.7 KYXC improves mitochondrial structure and function in mice

**FIGURE 7:**
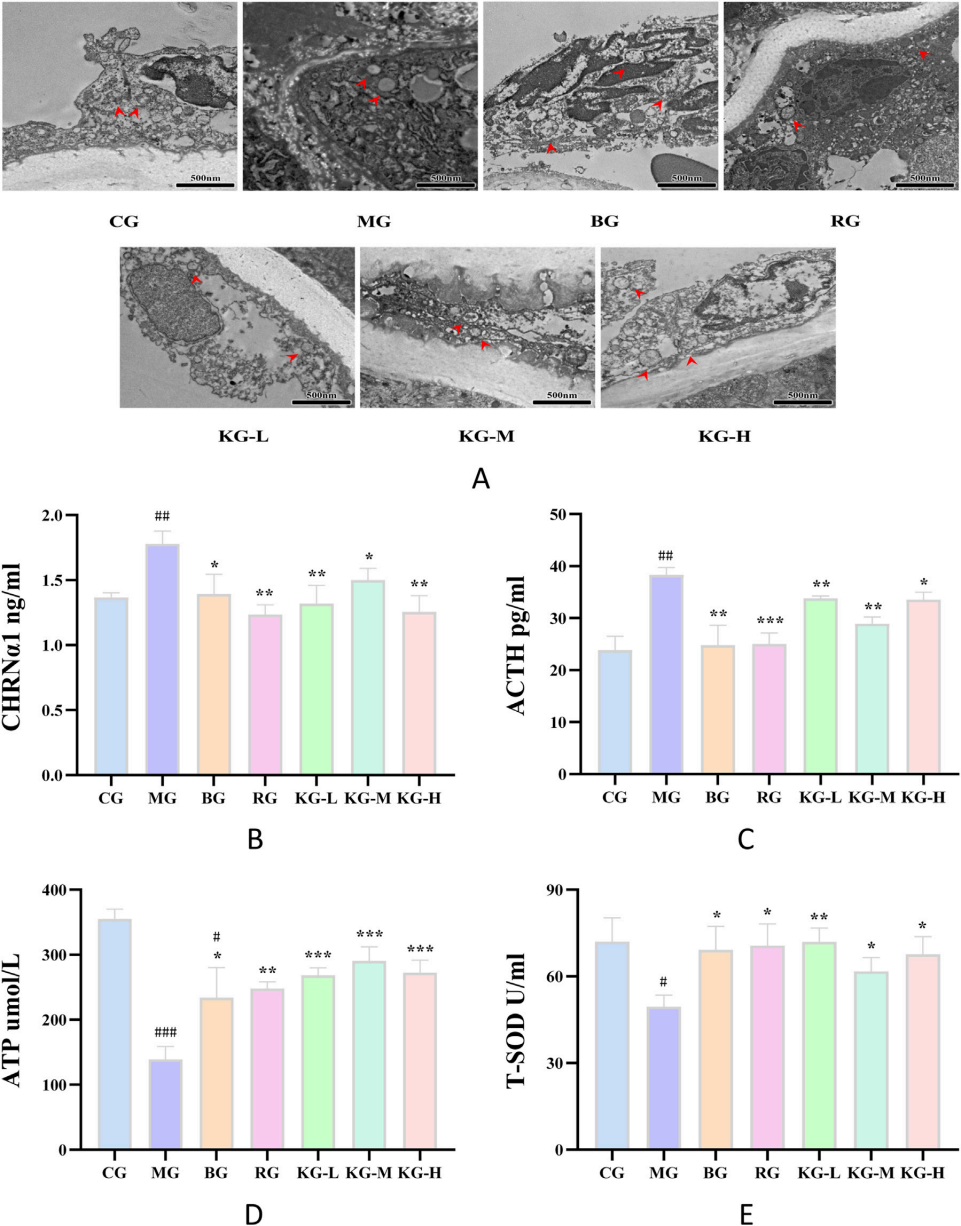
The effect of KYXC on the ultrastructure of mitochondria in AS mice. Scale bar: 500 nm **(A)** (The position indicated by the arrow in the figure is mitochondria) (Scale bar: 200 μm). The effect of KYXC on serum indicators of AS mice **(B–E)**. (Compared with the CG group, # *P* < 0.05, ## *P* < 0.01, ### *P* < 0.001; Compared with the MG group, * *P* < 0.05, ** *P* < 0.01, *** *P* < 0.001).

In order to further investigate the impact of KYXC on the aorta of AS mice, transmission electron microscopy was used to examine the mitochondrial ultrastructure of endothelial cells in each group of aorta. Figure 7A displays the findings from transmission electron microscopy. The mitochondria in the aortic endothelial cells of mice in the CG group displayed normal morphology, clear numbers, neat arrangement, and complete structure. In contrast, the endothelial cells of mice in the MG group exhibited a reduction or absence of mitochondrial cristae, severe damage to the membrane structure, and a significant presence of fat particles in their vicinity. The morphology of mitochondria in the RG, KG-L, KG-M, and KG-H groups of endothelial cells appeared to resemble the normal structure, with faintly visible mitochondrial cristae.

### 3.8 KYXC improves serum antioxidant levels in mice

According to the data presented in Figures 7B–E, the levels of ACTH and CHRNα1 were considerably higher in the MG group compared to the CG group (*P <* 0.01). After treatment, the RG, KG-L, KG-M, and KG-H groups all showed varied degrees of reduction in these levels (*P <* 0.05). In comparison to the CG group, the levels of ATP and T-SOD in the mice from the MG group were dramatically decreased (*P <* 0.05). Each treatment group exhibited a significant rise in these levels when compared to the MG group (*P <* 0.05). Furthermore, following the administration of different doses of KYXC, the ATP levels in the mice were shown to be higher compared to those in the RG group.

### 3.9 KYXC improves gene expression related to PGC-1α/Sirt3/Epac1pathway

**FIGURE 8:**
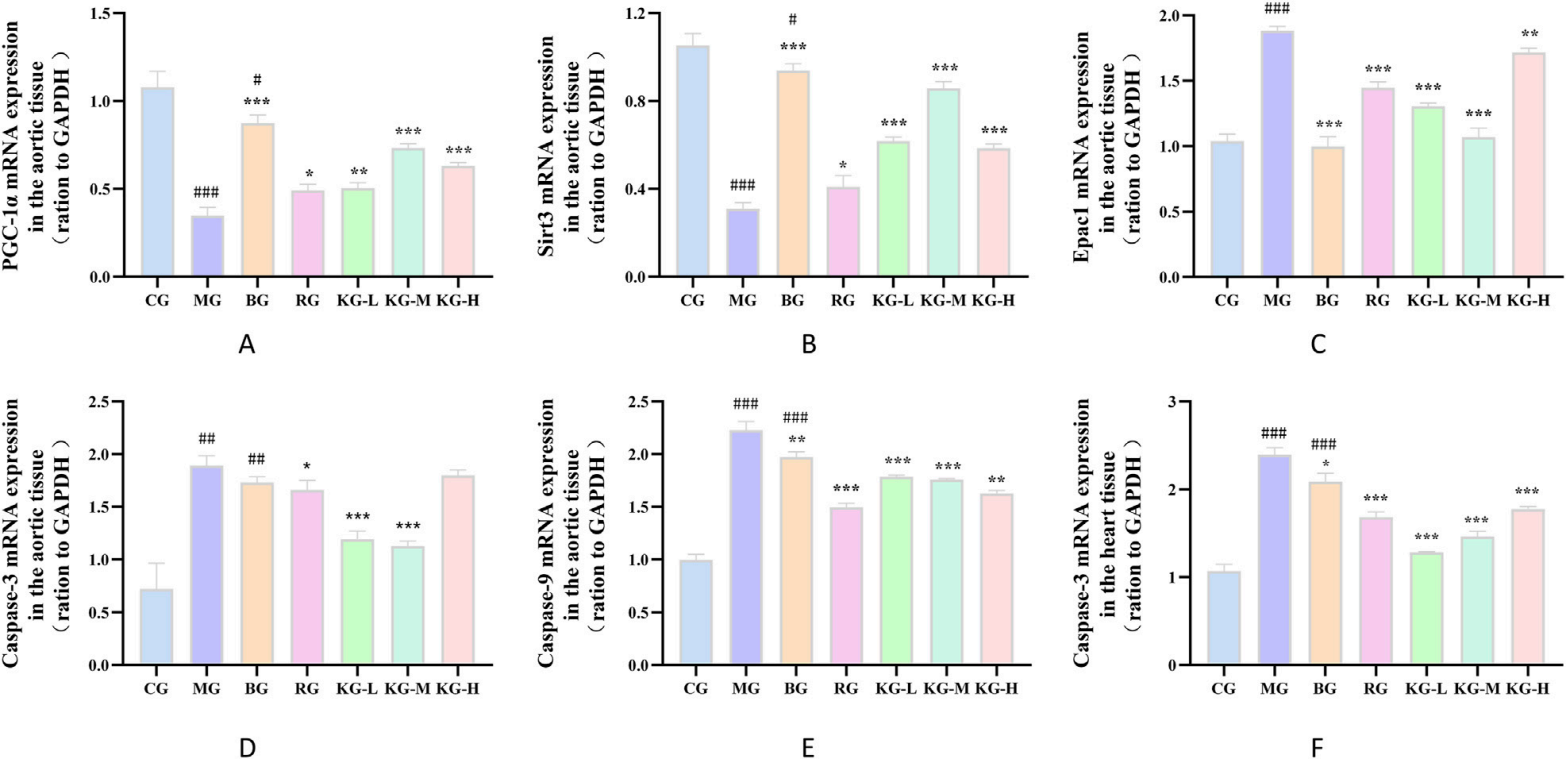
The effect of KYXC on the mRNA expression of PGC-1α, Sirt3, Epac1, caspase-9, and caspase-3 in the aortic tissue of AS mice **(A–E)**. The effect of KYXC on the mRNA expression of caspase-3 in the heart tissue of AS mice **(F)**. (Compared with the CG group, # *P* < 0.05, ## *P* < 0.01, ### *P* < 0.001; Compared with the MG group, * *P* < 0.05, ** *P* < 0.01, *** *P* < 0.001).

According to Figures 8A–E, the expression levels of PGC-1α and Sirt3 mRNA in the aortic tissue of mice in the MG group were significantly lower compared to the CG group. On the other hand, the expression levels of Epac1, caspase-9, and caspase-3 were significantly higher in the MG group (*P <* 0.01). When comparing the MG group to each treatment group, the expression levels of PGC-1α and Sirt3 mRNA in the aortic tissue were significantly higher, while the expression levels of Epac1, caspase-9, and caspase-3 mRNA showed varying degrees of improvement (*P <* 0.05). In the heart tissue, the expression level of caspase-3 mRNA was significantly higher in the MG group compared to the CG group (*P <* 0.01). However, in each treatment group, the expression level of caspase-3 mRNA in the heart tissue was reduced to varying degrees (*P <* 0.05), with no significant difference observed in other indicators. Refer to Figure 8F.

### 3.10 KYXC improves the expression of PGC-1α/Sirt3/Epac1 pathway related proteins and related apoptotic proteins

**FIGURE 9:**
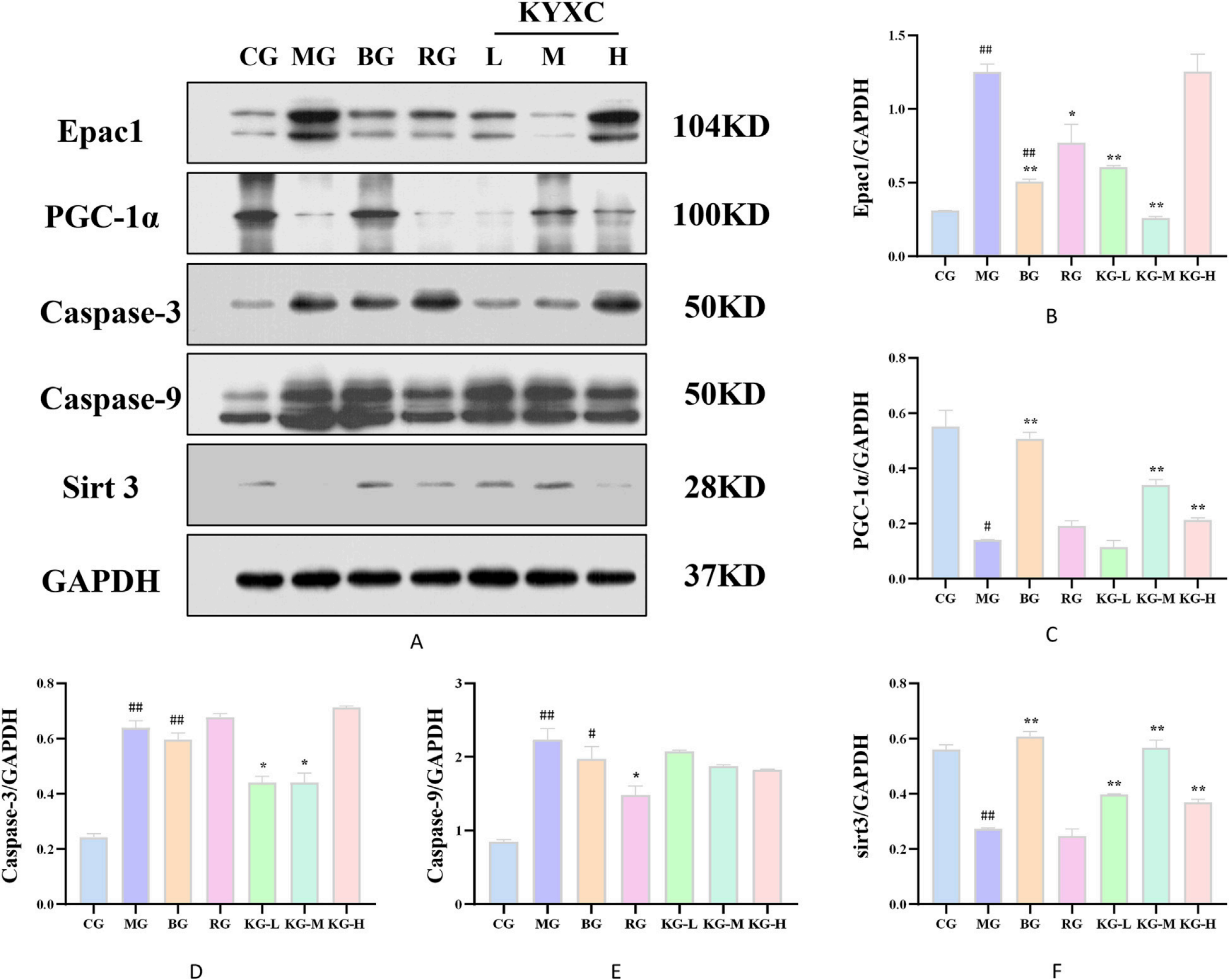
The effect of KYXC on the expression of PGC-1α, Sirt3, Epac1, caspase-9 and caspase-3 proteins in the aorta of AS mice **(A-F)**. (Compared with the CG group, # *P* < 0.05, ## *P* < 0.01, ### *P* < 0.001; Compared with the MG group, * *P* < 0.05, ** *P* < 0.01, *** *P* < 0.001).

Figure 9 displays the protein expression of mouse aortic tissue. In comparison to the CG group, the MG group showed a significant decrease in the expression levels of PGC-1α and Sirt3 proteins, while there was a significant increase in the expression levels of Epac1, caspase-9, and caspase-3 (*P <* 0.05). Furthermore, when comparing the MG group to each treatment group of mice, there was a significant increase in the protein expression levels of PGC-1α and Sirt3, while the protein expression levels of Epac1, caspase-9, and caspase-3 showed varying degrees of improvement (*P <* 0.05).

## 4 Discussion

As civilization has progressed and living conditions have improved, the prevalence of AS has steadily risen. Cardiovascular and cerebrovascular disorders resulting from atherosclerosis have emerged as the primary cause of mortality worldwide, garnering increasing attention from scholars (Roth et al., 2020). Atherosclerosis is strongly associated with dysregulation of lipid metabolism, inflammatory response, malfunction of vascular endothelial cells, and other underlying processes (Zhou and Wu, 2023). Researchers and academics have undertaken studies on frequently prescribed clinical medications that lower cholesterol levels, reduce inflammation, and prevent blood clotting (Wang et al., 2024). Although there has been notable advancement in drug-based treatment approaches, there are still persistent difficulties like drug resistance, adverse effects, and high rates of recurrence. Thus, it is crucial to strengthen treatment strategies in order to improve clinical efficacy.

Traditional Chinese medicine possesses the benefits of having various targets and routes as a result of its active substances that cause irritation. Consequently, it has demonstrated promise therapeutic efficacy in the treatment of cardiovascular illnesses. Several investigations have demonstrated that traditional Chinese medicine is capable of efficiently reducing the development of atherosclerotic plaque with few adverse effects. It has been widely employed in the management of cardiovascular disorders (Xing et al., 2020). Prior research has shown that valerian terpenes can enhance myocardial damage by suppressing the creation and function of xanthine oxidase, diminishing the synthesis of free radicals, mitigating the peroxidation of cell membrane lipids, limiting the aggregation of platelets, and enhancing microcirculation. Furthermore Nawrot et al. (2022), discovered through human pharmacology research that extracts derived from Valerian roots have the ability to significantly reduce heart rate in patients, providing protection to the heart and so enhancing the prevention and progression of cardiovascular diseases.

Nevertheless, there is currently a lack of complete study into the effects of KYXC on AS mice generated by a high-fat diet, and the precise mechanism by which it improves the AS effect remains unknown. This work effectively created AS model in ApoE^−/−^ mice by a high-fat diet. The objective was to examine the potential anti-atherosclerosis impact of KYXC and its underlying mechanism. The findings suggest that Volvalerenic A, the primary metabolite in KYXC, can enhance the accumulation of lipids, mitigate the inflammatory milieu, alleviate mitochondrial damage, and eventually impede the progression of atherosclerosis by activating the PGC-1α/Sirt3/Epac1 signaling pathway. Furthermore, this study utilized the cutting-edge technique of dynamic visualization of vascular microcirculation for real-time monitoring to visually assess the positive impact of KYXC on heart and mesenteric microcirculation. Following KYXC treatment, various parameters related to the vascular system, such as the vascular perimeter index, vascular diameter index, vascular skeleton density, vascular area density, and vascular complexity index, were found to have improved in both the heart and mesentery of mice. This suggests that KYXC has the potential to enhance arterial stiffness in these organs. The impact on membrane microcirculation more clearly demonstrates the significance of KYXC in enhancing microcirculation and cardiovascular function.

The presence of a lipid metabolism issue is regarded as the primary cause of AS and one of the most perilous elements contributing to the development of AS disorders (Manickam et al., 2022). This disorder is defined by abnormally high levels of cholesterol (CHOL) and triglycerides (TG) in the bloodstream (Kemal et al., 2020). Hence, assessing plasma CHOL and TG levels is a dependable method for evaluating lipid disorders. CHOL is an essential building block of cell membranes and is also a crucial metabolite of certain hormones, like steroid hormones. It plays a significant role in the transportation, metabolism, and storage of lipids (Lu et al., 2022). Thermogenesis is the primary energy source for the organism and also serves as a reservoir for surplus heat. Typically, the liver produces TG which is then carried by VLDL to peripheral tissues for lipid metabolism (Borén et al., 2022). However, elevated amounts of TG have a notable impact on the structural stability and thermal remodeling of VLDL and LDL (Jayaraman et al., 2019), resulting in the development of unstable plaques. As dyslipidemia progresses, the body’s ability to perform normal lipid metabolism and produce plaque is impaired, resulting in persistently elevated levels of CHOL and TG. The continuous buildup of lipids in atherosclerotic plaque speeds up the development of atherosclerosis. LDL is the main contributor to cholesterol and fat buildup on the artery wall (Malekmohammad et al., 2017). Following oxidative modification, LDL transforms into oxidized LDL (ox-LDL), which is the primary inflammatory metabolite of atherosclerotic plaque (Díaz-García et al., 2023). Subsequently, monocytes underwent a transformation into macrophages, which then engulfed ox-LDL through various mechanisms. This process resulted in the formation of foam cells, which led to the thickening of the inner layer of blood vessels (intima) and narrowing of the vascular lumen. These changes further exacerbated the development of atherosclerosis, ultimately resulting in the blockage of blood vessels and the formation of blood clots (thrombosis), along with other negative outcomes (Lu et al., 2022). VLDL, or very low-density lipoprotein, serves as the primary transporter of triacylglycerol in the bloodstream. Its levels tend to rise in certain medical disorders such diabetes, metabolic syndrome, obesity, and dyslipidemia (Jayaraman et al., 2019). Elevated triacylglycerol levels decrease the structural integrity of VLDL and its derivative LDL, hence initiating AS. The results obtained from the oil red O staining, HE staining, Masson staining, and biochemical analysis clearly demonstrate that KYXC effectively decreased the levels of CHOL, TG, and VLDL in the serum (*P <* 0.05). Additionally, KYXC reduced lipid deposition, inflammatory cell infiltration, and collagen fiber attachment in the aortic wall, thereby mitigating the development of vascular stenosis and unstable plaque, ultimately delaying the progression of atherosclerosis.

Mitochondria are organelles responsible for generating energy within eukaryotic cells. They are commonly referred to as the “power plants” of intracellular living activities and have significant involvement in lipid metabolism and energy metabolism (Liu et al., 2020). A primary role of mitochondria is to generate ATP, which serves as the energy source for numerous physiological processes in the body (Kim et al., 2016). Damage to the mitochondria can cause an imbalance in energy metabolism throughout the body, particularly in organs that have a high metabolic rate and require a lot of energy. Furthermore, mitochondria contribute to the regulation of cellular redox status by managing the generation and removal of reactive oxygen species (ROS) within the antioxidant defense system (Chen et al., 2024). According to Shuhong Yang et al. (2012), Valerian extract has been shown to dramatically decrease the levels of lactate dehydrogenase (LDH), creatine kinase (CK), and malondialdehyde (MDA), while significantly increasing the levels of superoxide dismutase (SOD), glutathione peroxidase (GSHPx), and ATPase. According to Wu and Zhang (2022), ACTH plays a role in lipid metabolism via controlling the mobility of mitochondria. Furthermore, during periods of bodily stress, there is a substantial rise in ACTH levels. This increase triggers the opening of mitochondrial membrane channel pores, resulting in a significant alteration of mitochondrial permeability. Consequently, the mitochondria experience swelling of the mitochondrial matrix and a loss of polarization in the mitochondrial membrane potential (Ran et al., 2024). These changes ultimately lead to the rupture of the outer membrane of the mitochondria, a decrease in ATP production, abnormal mitochondrial energy metabolism, and ultimately an impact on cellular function. Our research findings indicate that lipid metabolism irregularities lead to a substantial increase in the amount of ACTH (*P* < 0.01) and a significant drop in the level of ATP (*P* < 0.01). Additionally, these abnormalities disturb the structure of mitochondria and cause the disappearance of cristae. Following KYXC therapy, there was a reduction in ACTH levels and an improvement in ATP levels to different extents. This suggests that KYXC has the ability to enhance ACTH and ATP levels in the body, promoting homeostasis. The plasma membrane contains the entire amount of superoxide dismutase (T-SOD), which serves as an important indicator of oxidative stress in the body (Zheng et al., 2022). This study aimed to assess the degree of oxidative stress in the body by measuring the expression of T-SOD. As depicted in Figure 7E, the T-SOD content exhibited varying degrees of increase (*P* < 0.05) following KYXC treatment, suggesting a notable enhancement in the body’s oxidative stress level post-treatment.

Multiple studies have shown that the PGC-1α/Sirt3/Epac1 pathway is linked to cellular death. PGC-1α is a transcription regulatory factor that controls the body’s response to oxidative stress and mitochondrial metabolism (Zhang et al., 2020). Sirt3, the first deacetylase in the sirtuins family, is mostly present in the mitochondrial matrix and has a powerful protective effect on mitochondria (Li et al., 2020). Research has indicated that PGC-1α has a role in mitochondrial oxidative metabolism, cell apoptosis, and signal modulation by raising the expression of Sirt3 and enhancing deacetylase activity (Zu et al., 2021). Jia You et al. (2016) discovered that by over-expressing PGC-1α and reducing Sirt3 gene expression, they were able to demonstrate that PGC-1α can effectively enhance the expression of Sirt3, boost Sirt3’s deacetylase activity, and contribute to the regulation of myocardial hypertrophy, mitochondrial function, reactive oxygen species clearance, energy metabolism, and other related factors. Cyclic adenosine monophosphate (cAMP) is a ubiquitous second messenger found in cells that activates many proteins to control mitochondrial function. Epac1, a protein that is directly activated by cAMP, has demonstrated more effectiveness in controlling important factors such as MMP, ATP synthesis rate, and respiratory chain activity in mouse mitochondria (Li et al., 2024). Furthermore, the research conducted by Laudette et al. (2021) indicates that Epac1 has the ability to enhance the generation of mitochondrial ROS following extended lipid excess, while simultaneously decreasing mitochondrial respiration and ATP synthesis. Our study also showed that as the expression level of Epac1 protein decreased, the CHOL and TG contents in mice blood lipids significantly decreased, while the ATP and T-SOD contents in mitochondria significantly increased, which is consistent with previous research reports. The initiation of cellular apoptosis is marked or accompanied by an elevation in the concentration of apoptotic proteins. Stimulation of cells leads to the release of pro-apoptotic proteins, including cytochrome C. This release activates the caspase-9 and caspase-3 systems, with caspase-9 acting as an upstream promoter and caspase-3 as a downstream effector (Park, 2024; Peng et al., 2024). This work conducted RT-qPCR studies to examine the mRNA expression of PGC-1α, Sirt3 and Epac1 proteins, as well as their linked apoptotic proteins, before and after administration. The results revealed that KYXC can increase the expression of PGC-1α in AS mice. The mRNA expression levels of Sirt3, Epac1, Caspase-3, and Caspase-9 were decreased significantly (*P <* 0.05), which aligns with prior experimental findings. Thus, we posit that maintaining the proper functioning of the PGC-1α/Sirt3/Epac1 signaling pathway could be a crucial approach to treating AS.

However, our study exclusively examined the potential protective effects of *Valeriana officinalis L.* extract and its underlying molecular mechanisms using an animal model, rather than a cellular model of depression, which introduces certain limitations to the research. To determine whether *Valeriana officinalis L.* extract possesses potential for development as an anti-atherosclerosis drug, *in vitro* experiments are also imperative. Despite these limitations, our research provides valuable reference points for future studies.

## 5 Conclusion

The aforementioned findings suggest that *Valeriana officinalis L.* extract shows great potential as a Chinese herbal medicine for the management of atherosclerosis. The visualization of vascular microcirculation *in vivo* monitoring system and advanced bioinformatics technology are applied to validate the efficacy and mechanism of *Valeriana officinalis L.* extract in improving dyslipidemia in atherosclerotic mice. This was achieved by modulating the PGC-1α/Sirt3/Epac1 signaling pathway. Our findings aim to offer novel insights for the clinical management of atherosclerosis using modernized traditional Chinese medicine. This study has initially identified valtrate as the primary metabolite in the extract of *Valeriana officinalis L*. However, further investigation is required to establish whether Volvalerenic A is responsible for improving AS plaques and to identify any more potent metabolites. And the related study is going on in our lab.

## Data Availability

The original contributions presented in the study are included in the article/Supplementary Material, further inquiries can be directed to the corresponding authors.
