# Corrigendum: Mechanism of *Valeriana officinalis L*. extract improving atherosclerosis by regulating PGC-1α/Sirt3/Epac1 pathway

**DOI:** 10.3389/fphar.2025.1607975

**Published:** 2025-04-24

**Authors:** Bo Yao, Jingzhuo Ma, Qingzhi Ran, Hengwen Chen, Xuanhui He

**Affiliations:** ^1^ Guang’anmen Hospital, China Academy of Chinese Medical Sciences, Beijing, China; ^2^ School of Pharmaceutical Sciences, Changchun University of Chinese Medicine, Changchun, China

**Keywords:** *Valeriana officinalis L.*, extract, atherosclerosis, PGC-1α/Sirt3/Epac1 pathway, mitochondrial damage, traditional Chinese medicine

In the published article, there was an error in the **Funding** statement. Two important pieces of funding support [“the National Natural Science Foundation of China (No. 82205091)” and “Scientific and Technological Innovation Project of China Academy of Chinese Medical Sciences (CI2021A04619)”] were erroneously omitted. The correct **Funding** statement appears below.

